# Ministernotomy for correction of ventricular septal defect

**DOI:** 10.1186/s13019-016-0475-2

**Published:** 2016-04-26

**Authors:** Anh Tuan Vo, Thien Tam Vu, Dinh Hoang Nguyen

**Affiliations:** Cardiovascular Surgery Department, University Medical Center, University of Medicine and Pharmacy of Ho Chi Minh City, Ho Chi Minh City, Vietnam; 108B Nguyen Van Luong Appartment, Ward 12, District 6, Ho Chi Minh City, Vietnam

**Keywords:** Ventricular septal defect, ministernotomy, minimally invasive surgery

## Abstract

**Background:**

The development of minimally invasive surgery in the adult has created motivation for similar approaches in the congenital heart domain. Over the past 20 years, this type of surgery has been advocated in an effort to reduce costs related to hospital stay, and to improve the cosmetic results. We report our experience with ventricular septal defect repair utilizing a ministernotomy incision.

**Methods:**

From August 2014 to August 2015, 26 patients underwent ministernotomy for correction of ventricular septal defect at our center. All patients were between the ages of 14 months-old to 24 years-old with weight ranged from 7.5 to 54 kg (median weight 12 kg). Diagnoses were confirmed with echocardiography. We analysed in-hospital and 6 months follow-up outcomes of the group.

**Results:**

All defects were corrected successfully with satisfactory exposure. The median cardiopulmonary bypass time was 64 min, and median cross clamp time was 42 min. The intensive care unit stay ranged from 1 day to 3 days (median ICU stay, 1.5 days) and the hospital stay ranged from 4 to 13 days (median hospital stay, 5 days). There were no deaths during the operation or severe postoperative complications. No residual shunts were observed.

**Conclusion:**

Our results demonstrated the safety and efficacy of ministernotomy for the correction of ventricular septal defect with improved cosmetic results in patients greater than 7.5 kg. This aprroach can be used in either the transatrial or transarterial approach, and in smaller weight infants.

## Background

The introduction of minimally invasive surgery in the adult population has stimulated similar approaches in the congenital heart domain. Over the past two decades, this type of surgery has been advocated for both adults and children in an effort to reduce costs related to hospital stay, and to improve the cosmetic results. Particularly in the pediatric population, awareness should be paid to the cosmetic and psychological consequences of a conventional full sternotomy, as this could have an important role in postoperative recovery [3]. By using minimal skin incisions and a ministernotomy, surgical trauma can be reduced.

These techniques may necessitate costly equipment that can increase the cost of these procedures compared to that of conventional surgery. However, an inferior ministernotomy does not require any expensive equipment. This approach was first reported for correction of atrial septal defect [2]. As experience with ministernotomy in atrial septal defect repair developed, this approach has been applied to other congenital heart diseases, especially the ventricular septal defect.

sShould one encounter difficulties in exposure or performing the procedure, it is always feasible to convert to a full sternotomy. At our center, along with safe application of peripheral and central cardiopulmonary bypass and adequate myocardial protection, adequate exposure was achieved using a self-retaining retractor for the upper part of the incision.

The goal of our experience was to assess the efficacy and safety of using minimally invasive surgery in repairing ventricular septal defects in small children down to 8 kg in three types of cardiac malformations: the perimembranous VSD; the infundibular VSD; and the doubly-committed VSD.

## Methods

From August 2013 to August 2014, 26 patients underwent ministernotomy for repair of ventricular septal defects at the University Medical Center, University of Medicine and Pharmacy of Ho Chi Minh City, Viet Nam. Fifteen males and 11 females underwent operation with age varied from 14 month-old to 24 year-old (median age, 5 year-old). Patient’s weight ranged from 8 kg to 54 kg (median weight, 15 kg). The malformations corrected are summarized in (Table 1).

**Table 1:** Type of ventricular septal defects treated with a ministernotomy

Type of defect	Number of patients
Perimembranous VSD	17
Infundibular VSD	6
Doubly committed VSD	3
Total	26

### Operative techniques

**Fig. 1:**
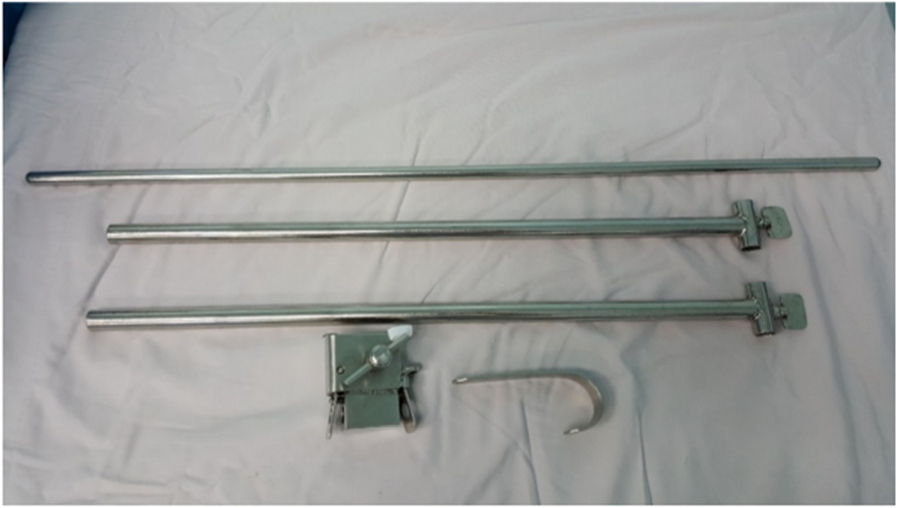
Self-retaining retractor, notice the small blade selected for a 14 kg patient

The patient is placed in the supine position with hands along the body as in conventional cardiac procedures. After induction with general anesthesia, transesophageal echocardiography (TEE) is routinely performed. After positioning and draping the patients, the self-retaining retractor (Fig. 1) is set up before incision at the top of the table. This retractor consists of blades in different sizes to fit the patients.

Before the ministernotomy, we expose the femoral vein, usually on the right side, using a small incision (1 cm) at the groin, and place a purse string on the vein. The femoral vein size is sufficient for inferior vena cava cannulation, and provides adequate venous return.

**Fig. 2:**
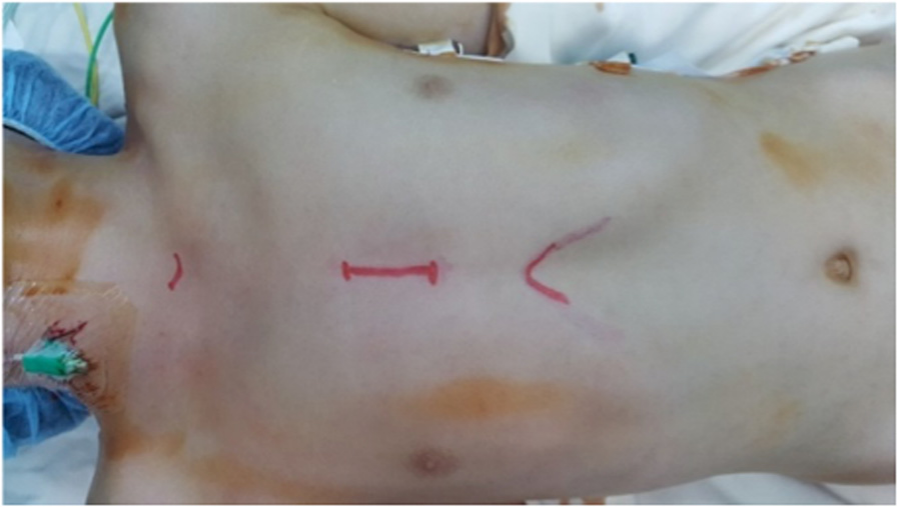
Skin incision

We perform a 3 – 5 cm skin incision, starting at the lower border of the manubrium of the sternum and extended to nipple line (Fig. 2). Subcutaneous tissue and the pectoralis major muscle are dissected using electrocautery. The sternum is divided in the midline and extended cephalad using an oscillating saw. The skin at the top of the incision is retracted using two Army/Navy retractors and the sternotomy is further extended using a strait Mayo scissors.

**Fig. 3:**
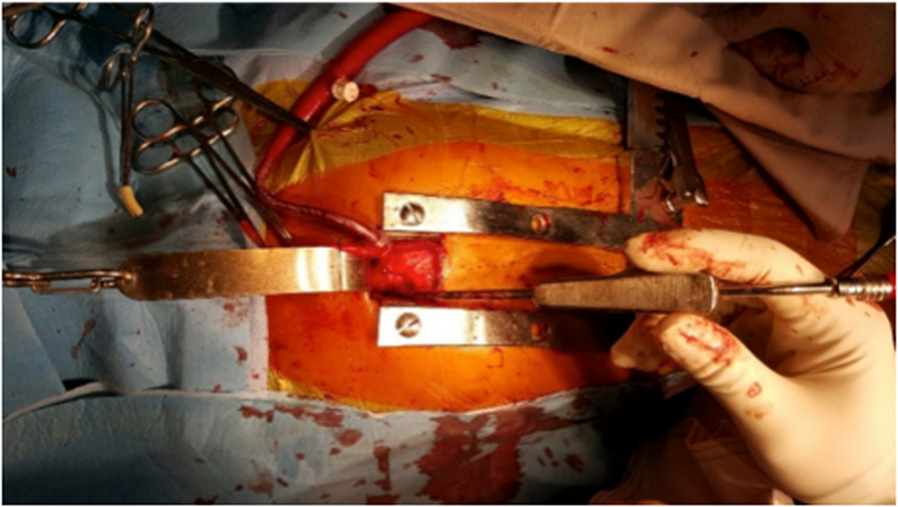
The operative field, notice the cephalad fastened self-retaining retractor facilitating the exposure of the great arteries

By using an inverse J sternotomy with the extension to the right border of the sternum, one can reduce the bleeding from bone fracture when retracting the sternum during the operation. A subtotal or total thymectomy is routinely performed, and several pericardial traction sutures are used to enhance the exposure of the aorta, the superior vena cava, as well as the pulmonary artery. Autologous pericardial patch is harvested and treated with glutaraldehyde. The ascending aorta is cannulated using a straight arterial cannula. The superior vena cava is cannulated directly, and the lower body venous return is drained by a long femoral cannula advanced from the groin. The femoral cannula is advanced just below the RA, and both vena cava are snared to restrict blood draining into the RA. Cannulation is facilitated by retracting the self-retaining retractor to further expose the aorta and the superior vena cava (Fig. 3).

**Fig. 4:**
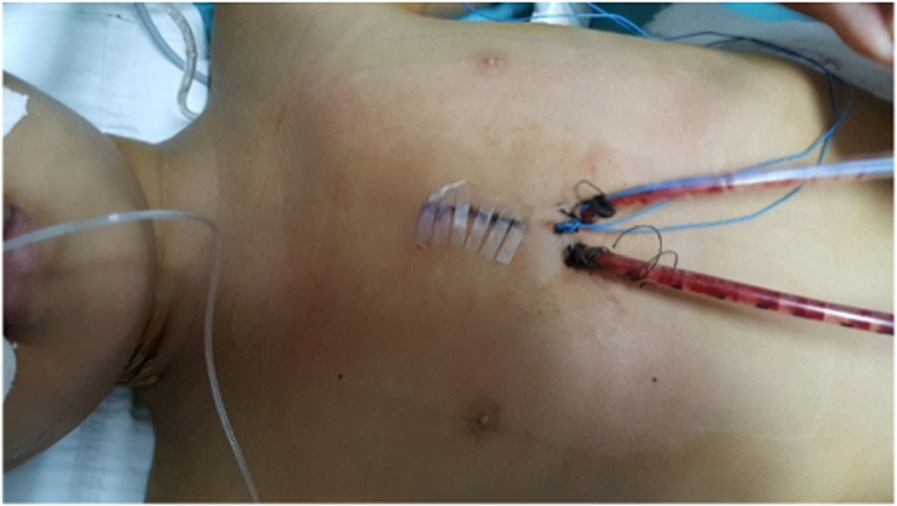
The skin incision after surgery

Once cardiopulmonary bypass (CPB) is started and adequate perfusion is achieved, the aorta is crossclamped, and cardioplegia is delivered antegrade through an aortic root needle. For the first 20 cases we used Custodiol cardioplegia. With experience with the new techniques, we changed back to 4:1 blood cardioplegia along with systemic cooling to 34 °C. The right atrium is opened and left ventricular venting is performed through the fossa ovalis. In the case of a perimembranous VSD, a conventional transatrial approach through the tricuspid valve is performed. By losing the self-retaining retractor, the lower part of the heart can be easily seen, thus providing adequate exposure of the surgical field. With a VSD requiring a transarterial approach, the self-retaining retractor is kept fastened thus providing a clear view of the great arteries. With the flexibility of the retractor, exposure of the upper part or the lower part of the heart can be done easily by loosening and tightening the retractor to fit the patient. Conventional techniques are utilized for the intracardiac VSD repair with continuous 5.0 prolene suture for > 10 kg patients, and continuous 6.0 prolene suture for 7.5 – 10 kg patients. TEE is used routinely to assess leaks in the VSD repair. Skin closure was performed intradermally (Fig. 4).

## Results

Patient demographics and postoperative data are summarized in Table 2. There were no deaths, or conversion to full sternotomy. Mean cardiopulmonary bypass was 77 ± 33.8 min, medium cross clamp time was 44 ± 20.1 min. The operating time ranged from 125 min to 320 min (median operating time, 198 min). Median ventilation time was 6 h. Intensive care unit stay varied from 1 to 3 days (median ICU time, 1.5 days). The hospital stay ranged from 4 to 13 days (median hospital stay, 5 days). No severe complications (residual shunt, aortic regurgitation and AV block) were recorded. There was one right pneumothorax requiring chest tube in one patient, and a large pericardial effusion requiring surgical drainage in one patient. There were no reoperations for bleeding or other reasons (Tables 2 and 3).

**Table 2:** Demographic and postoperative data

Parameters	Data
Male/Female	15/11
Age	14 month-old – 24 year-old (mean 5.6 ± 4.3)
Weight (kg)	7.5–54 (median, 12)
Operating time (min)	125–320 (median, 198)
ICU stay (days)	1–3 (median, 1.5)
Hospital stay (days)	4–13 (median, 5)

**Table 3:** Postoperative complications

Complications	Data
Death	0
Residual shunt	0
Aortic regurgitation	0
Heart block	0
Pneumothorax requiring chest tube	1
Pericardial effusion needing drainage	1

## Discussion

Since the first successful repair of an ostium secundum atrial septal defect by Gibbon in 1953, median sternotomy has been the gold standard approach to repair congenital cardiac conditions. However, over the past 3 decades, the minimally invasive surgery techniques have increased in cardiac surgery in the adult. The interest to introduce these techniques in pediatric population has also increasing, especially in lower weight groups [1]. Multiple articles regarding this topic have been published in the last 20 years (Table 4).

**Table 4:** Recently published results of minministernotomy VSD closure

Authors	Number of VSD	Year	Benefit		
			Cosmetic	Increased mortality/morbidity	Shorter in-hospital stay
Luo et al. [7]	66	2001	+	-	+
Da Silva et al. [3]	20	2006	+	-	Not mentioned
Sebastian et al. [9]	8	2009	+	-	Not mentioned
Vietes et al. [4]	24	2015	+	-	+

There are two reasons to change the approach for congenital heart disease from a conventional sternotomy to a minimal access sternotomy. First is the cosmetic aspect affecting the incision, postoperative pain, and possible negative consequences on pulmonary function. Second is the development of cardiopulmonary bypass, thus allowing safer peripheral cannulation without decreasing the venous return in the presence of the vacuum system [8].

Several recent articles have addressed the safety of ministernotomy for the repair of congenital heart defects. Luo et al. [7] reported a randomized prospective series of 100 patients with septal defects undergoing repair via ministernotomy or full sternotomy. Three advantages of ministernotomy were noted. First, there was decreased postoperative chest tube drainage. Second, hospital stay was shortened. Third, the procedure provided a better cosmetic effect, especially in young women [7]. However, apart from the cosmetic effect, the other benefits remained controversial. Laussen et al. [6] found no significant differences in terms of pain, emesis, pain drug requirements, respiratory rate, recovery or in-hospital stay in a group of 17 children undergoing ministernotomy for ASD repair, and compared with a group of 18 children undergoing full sternotomy. Nevertheless, with a larger series and several types of defects analyzed, Vieites et al. [4] reported ministernotomy had a higher early extubation rate and fewer complications than full sternotomy.

Sebastian et al. [9] found no differences in the number of blood transfusions but a prolonged cardiopulmonary bypass and cross-clamp times in the ministernotomy group. The issue of increased surgical time with minimally invasive surgery is encountered not only with ministernotomy, but also with other less invasive approaches [5, 8]. This has been explained by a smaller surgical field and a more complicated cannulation technique. However, the minimally invasive approach for VSD repair provides an excellent outcome with no increase in perioperative morbidity [4, 7].

Among the advantages of ministernotomy, the cosmetic effects remain the major benefit over the full sternotomy. Vietes et al. [4] found an improvement in cosmetic outcomes, as well as patient satisfaction after ministernotomy. In our study, with a smaller weight population, the limited skin incision could be retracted to provide improved exposure by losing or tightening the self-retaining retractor along with multiple pericardial traction sutures and the Army/Navy retractors. This gives the surgeon the flexibility to change from the upper half and the lower half of the operative field without extending the skin incision. Should difficulties in closing the VSD occur, transatrial approach and transarterial approach could be used at the same time to facilitate the operation. Furthermore, our results support the minimally invasive approach in lower weight patients.

Limitations of this study include the small number of patients and the lack of data regarding the level of pain, which is difficult and subjective to assess in pediatric patients.

## Conclusion

In conclusion, we have demonstrated the efficacy and safety of the ministernotomy to repair ventricular septal defects. This can be used in either the transatrial or transarterial approach, and in smaller weight infants. The small incision also provides very good results in cosmetic. There has been no difficulties in technical repair and no conversion to full sternotomy. The ministernotomy has become our preferred approach for children with ventricular septal defects, and weight > 7.5 kg.

### Ethics, consent and permissions

Written informed consent was obtained from all participants.
